# Plasma extracellular vesicles from recurrent GBMs carrying LDHA to activate glioblastoma stemness by enhancing glycolysis

**DOI:** 10.7150/thno.102014

**Published:** 2025-02-26

**Authors:** Xin Zhang, JunJie Li, Yiyao Huang, Anming Yang, Xiaoliu Liu, Yunhao Luo, Hao Tian, Minghui Wen, Chengzong Zhong, Bin Peng, Haitao Sun, Lei Zheng

**Affiliations:** 1 Department of Laboratory Medicine, Guangdong Provincial Key Laboratory of Precision Medical Diagnostics, Guangdong Engineering and Technology Research Center for Rapid Diagnostic Biosensors, Guangdong Provincial Key Laboratory of Single Cell Technology and Application, Nanfang Hospital, Southern Medical University, Guangzhou, 510515, China.; 2 Institution of Brain Diseases, Nanfang Hospital, Southern Medical University, Guangzhou, Guangdong, 510515, China.; 3 Department of Molecular and Comparative Pathobiology, Johns Hopkins University School of Medicine, Baltimore, MD, USA.; 4 Department of Neurosurgery, Nanfang Hospital, Southern Medical University, Guangzhou, China.; 5 Neurosurgery Center, The National Key Clinical Specialty, The Engineering Technology Research Center of Education Ministry of China on Diagnosis and Treatment of Cerebrovascular Disease, Guangdong Provincial Key Laboratory on Brain Function Repair and Regeneration, The Neurosurgery Institute of Guangdong Province Zhujiang Hospital, Southern Medical University, Guangzhou, China.

**Keywords:** GBM, LDHA-EVs, glioma stem cells, glycolysis, EV-based liquid biopsy, recurrence monitoring

## Abstract

**Rationale:** Glioblastoma multiforme (GBM) is the most aggressive primary malignant brain tumor in adults, characterized by high invasiveness and poor prognosis. Glioma stem cells (GSCs) drive GBM treatment resistance and recurrence, however, the molecular mechanisms activating intracranial GSCs remain unclear. Extracellular vesicles (EVs) are crucial signaling mediators in regulating cell metabolism and can cross the blood-brain barrier (BBB). This study aimed to elucidate how EV cargo contributes to the intracranial GSC state and validate a non-invasive diagnostic strategy for GBM relapse.

**Methods:** We isolated plasma extracellular vesicles (pl-EVs) from three groups: recurrent GBM patients post-resection, non-recurrent GBM patients post-resection, and healthy individuals. Newly diagnosed GBM patients served as an additional control. EVs were characterized and co-cultured with primary GBM cell lines to assess their effect on tumor stemness. EV cargo was analyzed using proteomics to investigate specific EV subpopulations contributing to GBM relapse. Based on these findings, we generated engineered LDHA-enriched EVs (LDHA-EVs) and co-cultured them with patient-derived organoids (PDOs). Metabolomics was performed to elucidate the underlying signal transduction pathways.

**Results:** Our study demonstrated that pl-EVs from recurrent GBM patients enhanced aerobic glycolysis and stemness in GBM cells. Proteomic analysis revealed that plasma EVs from recurrent GBMs encapsulated considerable amounts of the enzyme lactate dehydrogenase A (LDHA). Mechanistically, LDHA-loaded EVs promoted glycolysis, induced cAMP/ATP cycling, and accelerated lactate production, thereby maintained the GSC phenotype. Concurrently, post-surgical therapy-induced stress-modulated hypoxia in residual tumors, promoted LDHA-enriched EV release. Clinically, high levels of circulating LDHA-positive EVs correlated with increased glycolysis, poor therapeutic response, and shorter survival in recurrent GBM patients.

**Conclusion:** Our study highlights LDHA-loaded EVs as key mediators promoting GSC properties and metabolic reprogramming in GBM. These findings provide insights into recurrence mechanisms and suggest potential liquid biopsy approaches for monitoring and preventing GBM relapse.

## Introduction

Glioblastoma multiforme (GBM) is the most common primary malignant intracranial tumor in adults, characterized by extremely poor prognosis and high invasiveness 1. The standard treatment for GBM involves concurrent temozolomide (TMZ) with radiotherapy (RT) after resection, followed by 6 months of adjuvant TMZ 2. Even when glioma undergoes total resection at the macroscopic level 3, invasive tumor cells still exist in adjacent areas, making microscopic eradication of these cells impossible 3,4. These cells have been identified as glioma stem cells (GSCs), which possess significant self-renewal capacity and resistance to DNA-damaging modalities and appear to cause disease recurrence 5-8. Consequently, despite surgery, chemotherapy, and RT, almost all GBMs inevitably recur within 8-12 months post-operatively 6. Fast-growing GSCs typically exhibit increased glucose consumption, enhanced aerobic glycolysis, elevated lactate production (the "Warburg effect"), and higher ATP generation to maintain their stem-like properties 9-11. However, the underlying mechanisms and functional bio-molecules participating in GSC self-renewal remain unclear. Tracking post-operative residual GSCs status and validating functional molecules related to energy sources is critical for early warning of GBM relapse. Therefore, effective strategies need to be further developed.

Extracellular vesicles (EVs) are membrane-enclosed nanostructures released by cells that mediate intercellular communications and transport bioactive molecules to neighboring or distant cells 12-13. Due to their ability to cross the blood-brain barrier (BBB) 14 and disseminate bioinformation to distal organs 15, the relationship between peripheral EVs and intracranial GSC status post-operatively has become a subject of intense investigation in GBM recurrence. Clinically, elevated EV cargo concentrations have been found in the plasma of GBM patients, which decrease after surgery but rise again with tumor relapse 16. While previous studies have suggested that peripheral EV dynamics may indicate GBM status 12, the role of heterogeneous EV cargo in biofluids (such as cerebrospinal fluid, CSF, and plasma) in determining GSC status remains unclear. Moreover, since both the human brain and gliomas utilize high levels of glucose 17-18, and EVs have been recognized as crucial signaling mediators in regulating cell metabolism 19, the mechanism by which EV cargo induces specific metabolic signals has become a focal point of our research.

Lactate dehydrogenase A (LDHA) is a classical enzyme involved in anaerobic and aerobic glycolysis 20. As a sub-unit of LDH, LDHA preferentially converts pyruvate to lactate and NADH to NAD+ 21-22. Recurrent and advanced GBMs exhibit a ''Warburg phenotype'' with high lactic acid levels 7, 22. LDHA and lactate have recently been implicated as intracellular messengers, but further research on EV cargo-related glycolysis is required to determine its precise extracellular and intracellular roles.

In this study, we investigated the ability of plasma extracellular vesicle cargo as a biomarker for monitoring post-operative recurrence in GBM. Our findings demonstrated the abundance of LDHA-carrying EV subpopulation in the peripheral circulation of recurrent GBM patients. Furthermore, we provided evidence that LDHA-EVs activated aggressive GSCs by regulating glucose metabolism. Clinically, tumor recurrence after RT and/or chemotherapy following surgical resection increased LDHA loading into EVs, contributing to the pathological phenotype of intracranial GSCs. Monitoring LDHA-EV levels and interrupting LDHA-EV signaling may provide potential blood-based diagnostic and therapeutic strategies for GBM relapse.

## Methods

### Cell lines

Primary cell lines were isolated from tumor tissues of glioblastoma patients following the reported protocol 23, and were kind gifts from professor Songtao Qi's laboratory (Nanfang Hospital). U87-MG cells were transfected with a lentiviral vector encoding firefly luciferase reporter and the control vector, then selected by puromycin (2 µg/ml) for ~2 weeks to obtain U87-Luciferase (U87-MG-Luc) and U87-control (U87-MG-Ctrl).

### Glioblastoma organoids (GBOs) study

GBOs were prepared according to the method described by Nickl *et al*. 24. Patient-derived GBOs were fixed in 4% formalin (Carl Roth, Karlsruhe, Germany) for 24 h at 4 °C and used for immunohistochemical staining detected by Olympus VS200 ASW 3.2.1.

### Mouse model

Six-week-old female BALB/c nude mice were used to inject 2 × 10^5^ U87-GBM cells to establish a glioblastoma model *in situ*. Six mice were used in each group of experiments. Mice were divided into 3 groups after tumor injection and treated with various EVs, including HD-EVs, NR-EVs, and R-EVs (30 μg of EVs every 3 days via intra-peritoneal injection in PBS) for 5 different time points. Additionally, mice were divided into 3 groups after tumor injection and treated with NR-EVs, R-EVs and R-EVs+GNE140 (30 μg of EV every 3 days via intraperitoneal injection in PBS) for 5 independent time points. Tumor volume was detected by luciferase via *in vivo* imaging using IVIS Spectrum CT (PerkinElmer).

### Specimen collection

Our study was approved by the Ethics Committee of Nanfang Hospital (#NFEC-2022-056). 265 cases of GBM patients including 84 recurrent and 181 non-recurrent patients. The clinical information of the enrolling patients was listed in **Table S1**. The GBM patients underwent surgery followed by Stupp`s protocol-guided chemoradiotherapy, and 50 healthy individuals were included in our study, all specimens had confirmed pathologic diagnosis and were classified according to the 2021 World Health Organization Classification of Tumors of the Central Nervous System. Patients were excluded if they had concurrent malignancies, underwent only stereotactic biopsy, had received prior chemo/radiotherapy, or had incomplete medical records. Clinical data collection encompassed demographic characteristics, tumor-specific parameters, and molecular profiles including IDH mutation status and 1p/19q co-deletion. In this cohort, plasma samples were prospectively collected at a median of 3 months prior to scheduled magnetic resonance imaging (MRI) assessment, enabling temporal correlation between liquid biopsy findings and radio-logical features.

Fresh samples were immediately preserved in liquid nitrogen and 4% polyformaldehyde. Whole blood was collected in EDTA tubes, centrifuged at 1,880 g for 10 mins, transferred to new tubes, and centrifuged at 2,500 g for 10 mins at room temperature (RT) to minimize contamination by platelets, as described 25.

### Isolation of EVs

An OptiPrep™ Density Gradient Medium (BasalMedia, #R714JV) was used before loading the samples onto an SEC column. Six mL of plasma from every third individuals was layered on top of a 2 mL 50%, 2 mL 30%, and 2 mL 10% iodixanol working solution before centrifugation at 178,000 × g with ~13 ml (SW 41 Ti rotor, Beckman Coulter) for 2 h at 4 °C and combined to 1 sample (N = 1). Collected samples were prepared and qEV original SEC column (IZON, ICO70-13099) was pre-washed with 10~20 mL sterile PBS and 500 µL pre-treatment supernatant was loaded. Subsequently, PBS was used to eluate EVs. Each 0.5 ml effluent represented 1 fraction, and 7-10 fractions were collected using 0.2-μm-filtered PBS as the elution buffer, as previously described 25,26,27.

### Characterization of EVs

Formvar/carbon-coated copper grids (Ted Pella, Inc., Redding, CA, USA) were pretreatment before loading samples. The grids and samples were incubated for 15 mins, fixed sequentially in 2% paraformaldehyde and 2.5% glutaraldehyde, and contrasted in 2% uranyl acetate, as previously reported 25. EV morphology was characterized by JEM 1200 EX II transmission electron microscope (TEM) (JEOL Ltd., Tokyo, Japan). Particle concentration was analyzed by Nano Sight® nanoparticle tracking and Zetaview®system with detection threshold of 3. EVs were diluted in PBS before the analysis. Each sample was configured with a blue 488 nm laser and a high-sensitivity scientific complementary metal-oxide semiconductor (sCMOS) camera. At least 200 completed tracks were analyzed per video. Particles were tracked and quantitated, and data were analyzed by their sizes using NTA software V.3.4.

### EV labeling and administration

To visualize the target organ *in vivo*, EVs were labeled according to the manufacturer's instructions (Life Technologies, USA), with modifications. Briefly, 90 μg of EVs/group were incubated with Vybrant DID (1:1000 in PBS) in the dark for 15-20 min. The labeled EVs were washed with 50 ml of PBS, and centrifuged at 120,000 × g for 1.5 h to remove the excess dye. Next, the Vybrant DID-labelled EVs were injected into the tail vein of BALB/C nude mice (6 weeks old, n = 3 per group/time point, dosage per mouse: 30 μg of EV in 100 μl of PBS). PBS with/without Vybrant DID was used as the control. At 0.5 h and 12 h after EV injection, the mice and the harvested tissues were subject to *in vivo* and *ex vivo* imaging. Fluorescence intensity was determined using an *IVIS* Spectrum system.

### EV proteomics

The EV samples (~30 µg, N≥3) were collected, and proteomics was performed. Protein Discoverer (v2.3) was used to identify and quantify proteins. Raw data have been deposited with the Proteome X Change Consortium.

### ATP detection

ATP detection kit (Beyotime, #S0026) was used to determine ATP level for cells cultured in 6-well plates, 200 µL/well of lysates were added. Subsequently, the plates were centrifugd at 12,000 g for 5 mins at 4 °C, and the supernatants were harvested. For the tumor tissues, 150 µL of lysate per 20 mg of tissue was added, centrifuged at 12,000 g for 5 mins at 4 °C, and the supernatants were collected. The prepared lysates were operated according to the instructions, and the ATP level was measured on the multi-plate reader (SpectraMax i3x, Molecular Devices).

### Determination of extracellular acidification rate (ECAR)

The EACR assay kit (BestBio, BB-48311, China) and BBcellProbeTMP61 were used to detect ECAR of tumor cells and tissues after injecting with EVs following the manufacturer's protocols.

### Lactate measurement

Cells were harvested for each assay (initial recommendation = 2 × 10^6^ cells) and washed with cold PBS. The cell pellet was suspended in 4 × volumes of Lactate Assay Buffer (~200 µL) and homogenized by pipetting up and down a few times. Then, the cells were centrifuged for 2-5 mins at 4 °C at top speed in a cold microcentrifuge to remove any insoluble material. The supernatant was transferred to a clean tube. The endogenous LDH was removed from the sample using the Deproteinizing Sample Preparation Kit-TCA (ab204708). A commercial L-Lactate Assay Kit (Abcam, Cambridge, UK, ab65330) was used according to the manufacturer's instructions, and the absorbance OD (570 nm) was determined with a microplate reader (SpectraMax i3x, Molecular Devices).

### Lactate dehydrogenase A(LDHA) activity measurement

LDHA Activity Assay Kit (Solarbio, BC0680) was used to evaluate the conversion ability of NAD^+^ and lactate to pyruvic acid of the plasma-derived EVs, Ctrl-EVs, and LDHA^+^EVs following the manufacturer's protocols.

### Determination of specific EVs by exo-counter

In the current study, we used a bead antibody capturing system, Exo-counter (Sysmex), to isolate and calculate specific EV subpopulations coupled with capturing with CD9 beads. LDHA^+^CD9^+^EV, and S100A8/9^+^CD9^+^EV were two of the specific EV subpopulations selected by Exo-Counter (sysmex) with 12.5 µL plasma. In this system, EVs were captured in the groove of an optical disc coated with antibodies against the EV surface antigens. The EVs captured by using CD9 antibodies were labeled with LDHA- and S100A8/9-conjugated magnetic nanobeads, and the number of the labeled EVs was counted with an optical disc drive, as previous reported 28-29.

### IHC staining and score

The tumor samples of patients and the cranium from animals were fixed in 4% paraformaldehyde for 24-48 h, embedded in paraffin, cut into serial 4-µm-thick sections, and stained with hematoxylin and eosin (LEAGENE, DH0006-2, Beijing, China) for histological examination. The immunohistochemical staining was performed using the ZSGB-BIO PV-9000 kit (Beijing, China) as per manufacturer's instructions. The tissue sections from paraffin-embedded human GBM specimens and xenograft tissues were stained with specific antibodies or nonspecific IgG as a negative control. The stained tissue sections were examined and scored independently by two pathologists blinded to clinical parameters. The immunostaining levels were scored as 0 (negative), 1+ (weakly positive, light yellow), 2+ (moderately positive, yellowish brown), and 3+ (strongly positive, brown). 0 and 1+ indicated low expression, whereas 2+ and 3+ indicated high expression in tumor cells.

### Statistics

We performed statistical analysis by Student's t-test and ANOVA test to compare differences between multiple groups by analysis of variance; representative images were counted by image J software; data were considered statistically significant at *p* < 0.05. Pearson correlation analysis was used to analyze correlation. All statistical methods were performed by using GraphPad Prism 8.3.0.

## Results

### Plasma EVs from recurrent GBMs promote GSC formation *in vitro*

**Figure 1:**
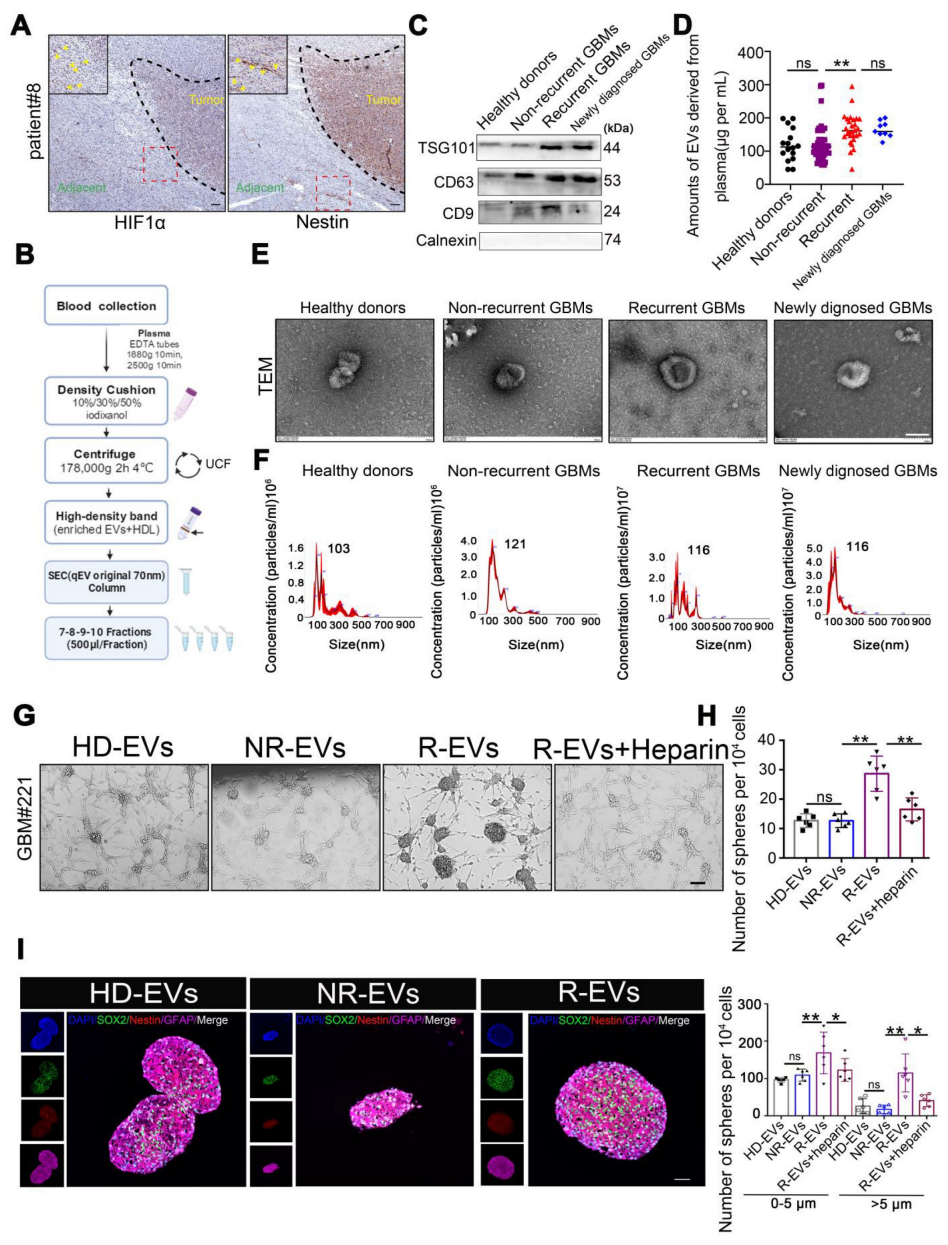
** Plasma EVs from recurrent GBMs promote GSC formation *in vitro*.** (A) IHC staining of Nestin positive and HIF-1α positive cells in the adjacent tissues from individual GBM patients#8. Scale bars, 100 µm. (B) Schematic overview of EVs separation from plasma. HDL, high-density lipoprotein, IDC, iodixanol density cushion, SEC, size exclusion chromatography, UCF, ultracentrifugation. (C) Immunoblot characterization of 30µL (~10µg) EVs with antibodies against the common EV markers (CD63, CD81, CD9) and cellular contaminants marker (Calnexin) on EVs from fractions 7-8-9-10, (500µL/fraction). (D) BCA quantification of pl-EVs amounts of combined 7-10 fractions. Healthy donors, HD, N = 16, Non recurrent, N = 60, recurrent GBM, R, N = 28, newly diagnosed GBMs, N = 9. (E) 30µL (about ~10µg) EVs evaluated with transmission electron microscopy and indicating the EV-like structures (cup-shaped). Scale bars, 100 nm. (F) Size distribution of 15µL pl-EVs obtained by NTA. (G) GSCs formation, 24 hours after ~20,000 GBM cells adhered, adding 5µg pl-EVs samples/well and incubated with/without 2µg/ml heparin, spheroids observed continued for 1 week at 37 ℃. Cells were culture with EVs depleted DMEM. Scale bars, 200 µm. (H) (up) Statistics of GSCs-like spheroids size, estimated using Spheroid Sizer. (down) Statistics of GSC-like spheroids in different sizes (**, *p* < 0.01, ns.non-significance). (I) immunofluorescence of cells by using anti-SOX2 (red), anti-Nestin (red), nuclei stained with DAPI (blue), and EVs labeled by PKH67 (green). Scale bar, 20µm.

Tissue sections from GBM patients exhibited Nestin- and HIF-1α-positive cells in the peritumoral region (**Figure 1A**), suggesting the potential existence of GSCs. We investigated the role of circulating EVs in modulating GSC cells by examining the interaction between EVs isolated from GBM patients' plasma (pl-EVs) and primary GBM cells in vitro. As detailed in the schematic diagram (**Figure 1B**), we isolated high-quality EVs from plasma samples of healthy donors (HD-EVs), non-recurrent GBM patients (NR-EVs), recurrent GBM patients (R-EVs), and newly diagnosed GBM patients (P-EVs). Immunoblotting confirmed the expected presence of EV-associated proteins (CD9, CD63, TSG101) while cellular contaminant markers (Calnexin) were undetected (**Figure 1C**). Additionally, bicinchoninic acid assay revealed that R-EVs and P-EVs contained significantly higher protein cargo than HD-EVs and NR-EVs (**Figure 1D**, *p* < 0.01). TEM and NTA revealed characteristic cup-shaped morphology and size distribution (30-250 nm in diameter) of all types of EVs (**Figure 1E-F**).

We investigated the potential role of EVs, by co-culturing primary GBM cells grown in an EV-depleted medium (confirmed by TEM, **Supplementary Figure 1**) with all pl-EVs (HD-EVs, NR-EVs and R-EVs) for one week. We observed that R-EVs significantly induced sphere formation (*p* < 0.01), which was attenuated by co-incubation with heparin (2 μg/μL, *p* < 0.01; heparin inhibits EV uptake, **Supplementary Figure 2**), compared to NR-EVs and HD-EVs. Quantification of spheres in defined fields indicated an increase in both small (0-5 μm) and large (> 5 μm) spheres following R-EVs treatment (**Figure 1G-H**). Furthermore, immunofluorescence analysis revealed increased expression of stem cell markers (Nestin and SOX2) in R-EV-treated spheroids (**Figure 1I-J**). These findings suggest that plasma-derived EVs from recurrent GBM patients promote GSC-like phenotypes in primary GBM cells *in vitro*.

### Plasma EVs from recurrent patients accumulate intracranially, inducing GSC phenotype and proliferation *in vivo*

**Figure 2:**
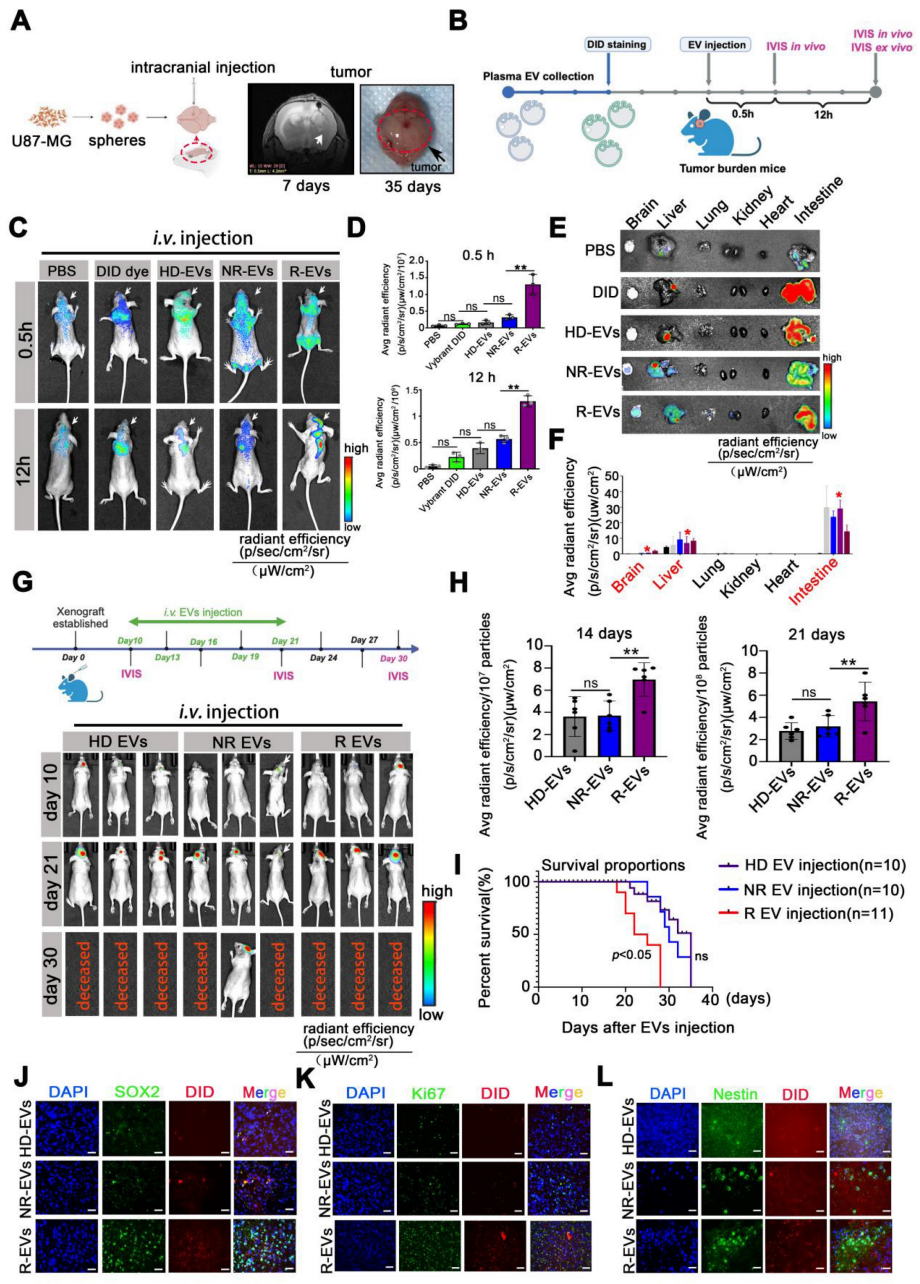
** Plasma EVs from recurrent patients accumulate intracranially, inducing GSC phenotype and proliferation *in vivo*.** (A) Experimental design of U87-MG intracranial model. U87-MG spheroids implanted into the nude mice following MRI imaging at 7 days and sacrificed at 35 days. (B) Experimental design of PBS, Vybrant-DID (1,1'-dioctadecyl-3,3,3',3'-tetramethylindodicarbocyanine perchlorate), and 30 µg/mouse Vybrant-DID labeling EVs (HD-EVs, NR-EVs and R-EVs) were* i.v.* injected and IVIS was detected at 0.5 h and 12 h *in vivo* and *ex vivo.* (C) Representative images of EV fluorescence at 0.5 h and 12 h. (D) Statistics of intracranial fluorescence at 0.5 h and 12 h. (E) biodistribution of DID labeling EVs in different organs (brain, liver, lung, kidney, heart and intestine) at 12 h. (F) Statistics of fluorescence area of DID label EVs in different organs. Red* indicated the radiant efficiency. (G) Experimental design of EVs injection with U87 MG-bearing mice and representative images of intracranial tumor with EVs injection at 10, 21, 30 days. (H) Statistics of tumor burden at 14 days and 21 days. n = 6. (I)Survival of U87-MG bearing mouse after HD-EVs (n = 11), NR-EVs (n = 10) and R-EVs (n = 11) administration. (J-L) Immunofluorescence staining of intracranial tumor tissues by using anti-SOX2 (green), anti-Nestin (green), nuclei stained with DAPI (blue), and EVs with DID dye (red). Scale bar, 10 µm. Data are presented as means ± SD.; *, *p* < 0.05; **p < 0.01; ***p < 0.001; ns., non-significance.

To evaluate the biodistribution of pl-EVs, we established an orthotopic U87-MG glioblastoma model and administered EVs following the protocol illustrated in **Figure 2A-B**. The emission from the skull area of mice administered with R-EVs was obviously observed at 0.5 h post-injection, compared to the other group (**Figure 2C**). Importantly, R-EVs significantly exhibited abundance in the brain region at 12 h after EV injection (**Figure 2C-D**), suggesting a potentially higher brain-targeting capability of R-EVs than other groups. To further validate the tumor-targeting efficiency, we established an intracranial xenograft model using U87 cells stably expressing GFP and administered 30 μg of HD-EVs, NR-EVs, or R-EVs. Analysis of brain sections harvested 12 h post-injection demonstrated that R-EVs exhibited superior tumor-specific accumulation compared to other groups (**Supplementary Figure 3**), indicating enhanced tumor-targeting capabilities.

*In vivo* imaging revealed that R-EVs demonstrated significantly enhanced accumulation in the cranial region at 0.5 h post-injection compared to control groups (**Figure 2C**). Notably, R-EVs maintained higher retention in the brain tissue at 12 h post-administration (**Figure 2C-D**). Besides, we also measured the *ex vivo* fluorescent from various soft organs (heart, liver, spleen, kidney, and intestine) harvested at 12 h and found no difference between the groups. At 12 h, the average fluorescence intensity in the liver and intestine in the groups was elevated substantially (**Figure 2E-F**). Furthermore, at 12 h post-injection, no pathological changes were detected in the soft organs of the groups, suggesting almost no systemic toxicity of the EVs (**Supplementary Figure 4**).

We developed an orthotopic model using luciferase-expressing U87-MG cells (U87-MG-Luc) to investigate the impact of pl-EVs on tumor progression. HD-, NR-, and R-EVs were administered intravenously on days 10, 13, 16, 19, and 21 post-tumor implantation (**Figure 2G**). R-EV administration significantly promoted tumor growth at 14 (*p* < 0.01) and 21 days (*p* < 0.01) post-tumor implantation compared to HD-EV and NR-EV groups (**Figure 2G-H**), and markedly reduced survival of mice over a ~35-day observation period (*p* < 0.05) (**Figure 2I**). Immunofluorescence of brain tissue revealed extensive localization of DiD-labeled R-EVs, accompanied by increased expression of stem cell markers (SOX2, Nestin) and the Ki67 proliferation marker in the tumor region (**Figure 2J-L**) and associated statistics indicated the Ki67, SOX2 and Nestin area to DAPI (%) (**Supplementary Figure 5**). These findings suggested that R-EVs induce an aggressive glioma stem cell-like phenotype and enhance proliferation rates *in vivo*.

### LDHA⁺CD9⁺EV subpopulations become prominent with GBM relapse

The EV-encapsulated proteins are involved in specific cellular functions under various physiologic and pathological conditions 12. Liquid chromatography-tandem mass spectrometry (LC-MS/MS) was carried out on equal amounts of HD-EVs (N = 3), NR-EVs (N = 4), and R-EVs (N = 4) from GBM patients to determine the protein components of isolated EVs.

**Figure 3:**
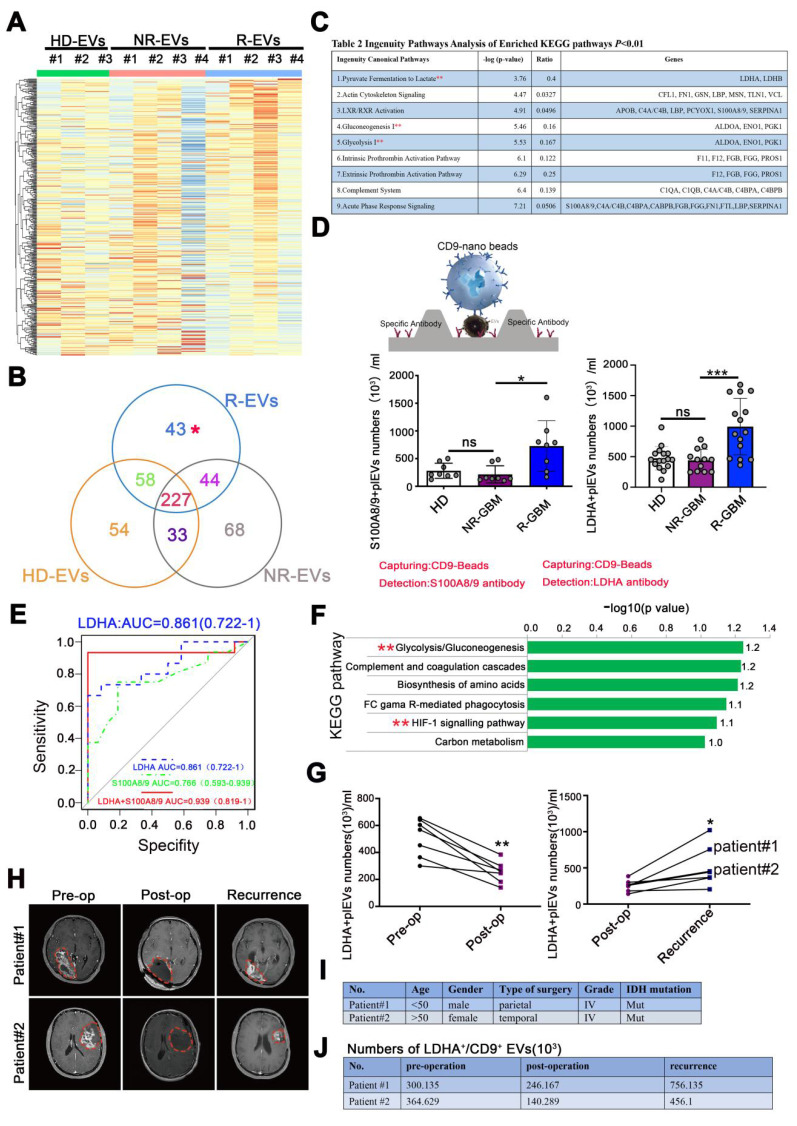
** LDHA⁺CD9⁺EV subpopulations become prominent with GBM relapse.** (A) LC-MS/MS analysis of the protein composition of EV sample and heatmap for plasma EV proteomic, EV sample: N ≥ 3. (B) Venn diagram. (C) main pathways and proteins changed in R-EVs analyzed by KEGG. (D) Schematic overview of Exo-counter capturing specific EVs using CD9 nano-beads and antibody & Numbers of S100A8/9^+^/CD9^+^ pl-EVs and LDHA^+^/CD9^+^ pl-EVs calculated by Exo-counter in 12.5 µL plasma. (E) ROC curve of S100A8/9^+^/CD9^+^, LDHA^+^/CD9^+^ and S100A8/9^+^ LDHA^+^/CD9^+^ pl-EVs to distinguish non-recurrent from recurrent GBMs. (F) ingenuity enriched KEGG up-regulated pathways specific to R-EVs. (G) numbers of LDHA^+^CD9^+^ pl-EVs calculated by exo-counter at pre-operation (pre-op), post-operation (post-op) and recurrence. (H) MRI images of GBM patients#1 & #2 with pre-op, post-op, and recurrence. (I-J) Patient characteristics and numbers of LDHA^+^ pl-EVs calculated by exo-counter in GBM patients#1 & #2. Data are presented as means ± SD. *, *p* < 0.05; **p < 0.01; ***p < 0.001; ns., non-significance.

R-EVs displayed a distinct protein profile compared with HD-, and NR-EVs (**Figure 3A-B**). We next performed a Kyoto Encyclopedia of Genes and Genomes (KEGG) analysis on proteins among the 3 groups. The data showed the enrichment of 43 distinct proteins in R-EVs in "pyruvate fermentation to lactate", "glycolysis", "gluconeogenesis", and 9 other signaling pathways (**Figure 3C, Table S2**). Also, typical proteins that were more abundant in R-EVs mainly clustered in glycolysis and hypoxia signaling pathways (**Figure 3F**). Intriguingly, LDHA, a classical enzyme involved in anaerobic and aerobic glycolysis, was significantly up-regulated 2.381-fold (**Figure 3C, Table S2**). Furthermore, Exo-counter, a highly sensitive EV counting system, allows the identification of specific EVs by utilizing optical disk technology and introducing nanobeads for EV capturing 28-29. Exo-counter can detect specific EVs derived from human plasma without any enrichment procedures and its detection sensitivity and linearity are higher than those of conventional detection methods such as ELISA or flow cytometry.

We further validated the typical EV sub-population by using CD9 beads and antibodies to quantify EVs 29. Our data demonstrated a significant increase in LDHA^+^/CD9^+^EVs (*p* < 0.001) and S100A8/9^+^/CD9^+^EVs (*p* < 0.05) in the plasma of recurrent GBM patients, compare to the NR and HD group (**Figure 3D**). Data from protein atlas indicated that both proteins exhibited prominent assembly on the plasma membrane and intracellular vesicles. Receiver operating characteristic (ROC) analysis revealed that LDHA^+^/CD9^+^EVs and S100A8/9^+^/CD9^+^EVs exhibited favorable diagnostic potential for distinguishing between recurrent and non-recurrent GBM patients post-surgery. The combination of both markers yielded an area under the curve (AUC) value of 0.939, indicating higher diagnostic accuracy (**Figure 3E**). However, in a paired clinical patient cohort, we observed that LDHA^+^/CD9^+^EV decreased markedly post-surgery (*p* < 0.01) and increased significantly with recurrence (*p* < 0.05) (**Figure 3G-H**). Detailed analysis of two individual GBM patients with post-surgical recurrence further corroborated the potential value of LDHA^+^EVs as a recurrence biomarker (**Figure 3I-J, Supplementary Figure 6**). These findings suggested that LDHA-positive EVs may serve as a candidate biomarker for monitoring GBM recurrence, complementing current imaging methods.

### Radio chemotherapy modulates hypoxia to drive LDHA-enriched EV release in glioblastoma

**Figure 4:**
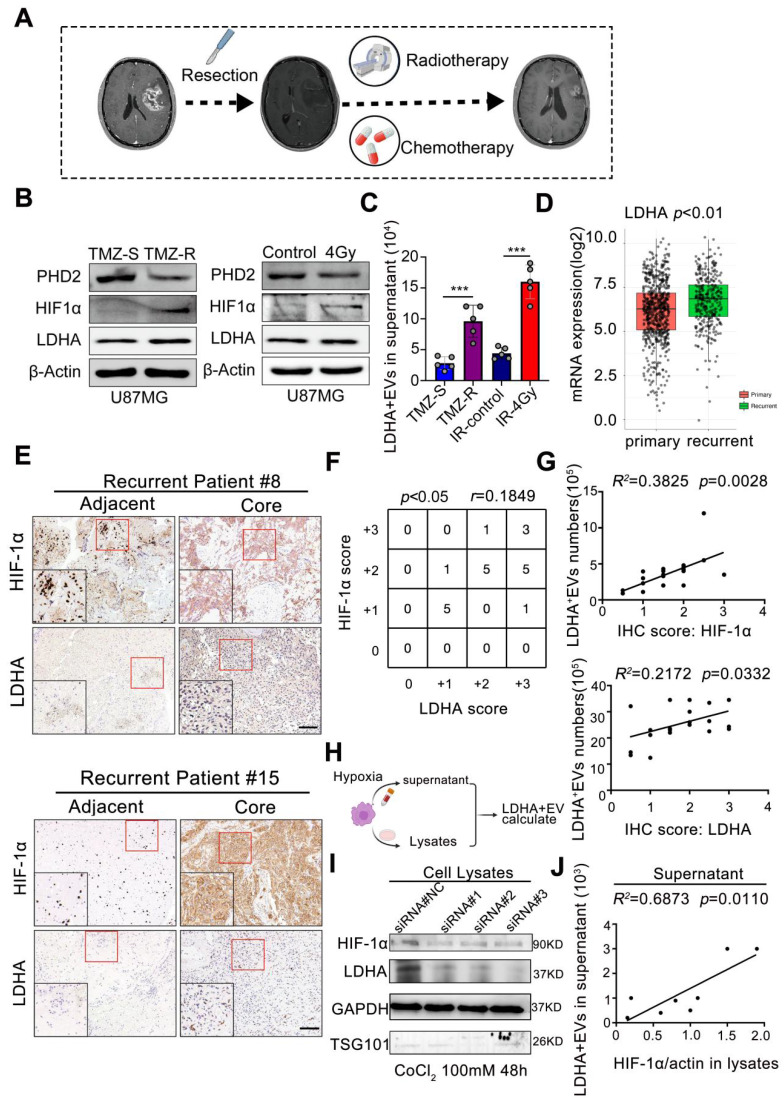
** Radio chemotherapy modulates hypoxia to drive LDHA-enriched EV release in glioblastoma.** (A) The current standard treatment for GBM was concurrent TMZ with IR after resection, and then followed by 6 months of TMZ. (B) The protein levels of PHD2, HIF-1α and LDHA in TMZ resistant and sensitive U87-MG cells and U87-MG treated with/without 4Gy radiotherapy. (C) number of LDHA positive EV in the supernatant of distinct groups (n = 5). (D) mRNA level of LDHA in primary and recurrent GBM patients analyzed by TCGA database. (E) Immunohistochemical analysis of HIF-1α, LDHA in adjacent and core tissues of recurrent GBM patients. (F) Correlation between the expression levels of HIF-1α and LDHA was assessed in GBM tumor tissues (n = 21). (G) Correlation between the expression levels of HIF-1α / LDHA in the tissues and LDHA positive EV numbers in the plasma of paired recurrent GBM patients (Chi-squared test and Spearman rank correlation test was used, respectively). (H-I) HIF-1α, LDHA levels in the cells that HIF-1α were induced by CoCl_2_ and silenced by siRNA. (J) The correlation between LDHA positive EV numbers in the supernatant and HIF-1α / LDHA level in the cell lysates, n = 8 (Chi-squared test and Spearman rank correlation test was used, respectively) (*, *p* < 0.05; **p < 0.01; ***p < 0.001; ns., non-significance).

We investigated the origin of the EV subpopulation to elucidate the mechanisms underlying Elevated peripheral LDHA-enriched extracellular vesicles (LDHA-EVs) during tumor recurrence. The current standard treatment for GBM consists of concurrent temozolomide (TMZ) with RT after maximal safe resection, followed by 6 months of adjuvant TMZ 2, as illustrated in **Figure 4A**. STRING analysis identified HIF-1α as a key gene in the LDHA-related network (**Supplementary Figure 6**). To model treatment resistance, we exposed U87-MG cells to TMZ treatment to generate TMZ-resistant and TMZ-sensitive cell lines, which were then subjected to RT (4Gy). Under normoxic conditions, HIF-1α is rapidly hydroxylated by prolyl hydroxylase domain 2 (PHD2) and subsequently undergoes ubiquitin-mediated proteasomal degradation. Immunoblotting analysis revealed that PHD2 was downregulated, whereas HIF-1α and LDHA were markedly upregulated in TMZ-resistant and irradiated cells (**Figure 4B**). Additionally, Exo-counter analysis demonstrated a significant increase in LDHA^+^ EVs in the cell culture supernatant of both TMZ-resistant (*p* < 0.001) and irradiated (*p* < 0.001) cells, indicating that radio-chemotherapy positively correlates with increased extracellular LDHA^+^EV release (**Figure 4C**).

The TCGA dataset revealed significantly higher LDHA expression levels in clinical samples from recurrent GBM patients than primary GBM patients (*p* < 0.01, **Figure 4D**). Furthermore, a positive correlation (r = 0.1849, *p* < 0.05) between HIF-1α and LDHA expression levels was observed in recurrent core tissues, as evidenced by immunohistochemistry (IHC) scores in paired GBM tissue samples (**Figure 4E-F**). The number of LDHA^+^/CD9^+^ EVs in plasma positively correlated with both HIF-1α scores (R² = 0.3825, *p* = 0.0028) and LDHA levels (R² = 0.2172, *p* = 0.0332) in paired patient samples (**Figure 4G**).

Hypoxia was induced by CoCl_2_ treatment in U87-MG cells (**Supplementary Figure 7**) to further confirm whether HIF-1α promoted LDHA sorting into EVs and the released LDHA-positive EVs were quantified using an Exo-counter (**Figure 4H**). Small interfering RNAs (siRNAs) targeting distinct sequences were utilized for HIF-1α silencing. The results indicated that hypoxia significantly increased both HIF-1α and LDHA expression while HIF-1α silencing markedly reduced their levels (**Figure 4I**). Exo-counter analysis confirmed that extracellular LDHA^+^ EVs positively correlated with intracellular HIF-1α levels (R² = 0.6873, *p* = 0.0110) (**Figure 4J**), demonstrating that HIF-1α stability was a critical regulatory factor promoting LDHA enrichment in released EVs. These findings demonstrated that radio-chemotherapy-induced hypoxia and enhanced HIF-1α stability significantly correlate with LDHA enrichment in circulating EVs.

### LDHA-enriched extracellular vesicles promote stemness in GBOs

**Figure 5:**
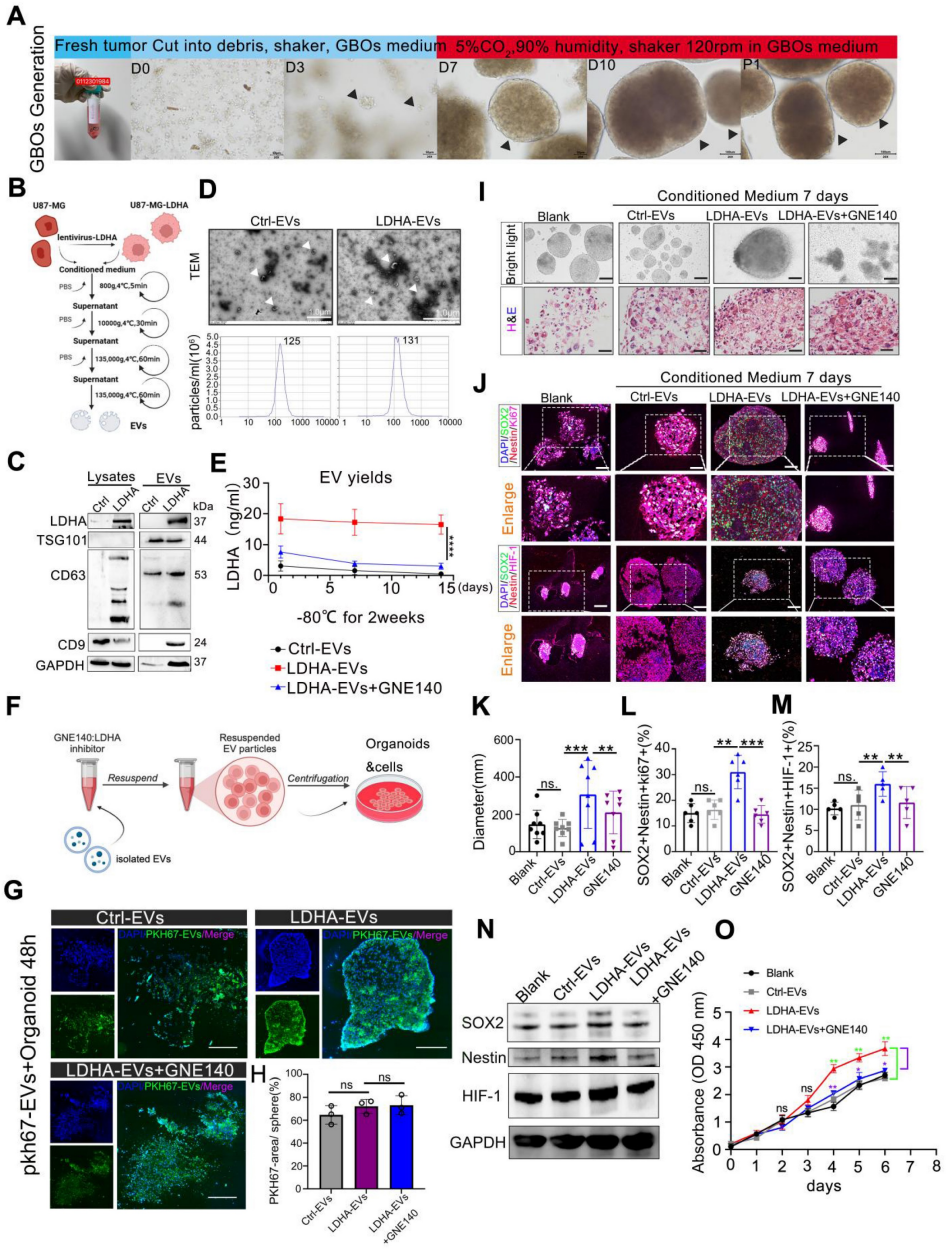
** LDHA-enriched extracellular vesicles promote stemness in GBOs.** (A) Workflow of GBOs generation. Scale bars: D0-D3, 50 µm, D7-P1, 100 µm. (B) workflow of engineered EVs and EVs concentration from supernatants. (C) Western blotting of presence of LDHA and EV-associated (TSG101, CD63, CD9) markers in LDHA-EVs and Ctrl-EVs. (D) TEM and NTA analysis of Ctrl-EVs and LDHA-EVs. Scale bars: 1µm. (E) LDHA concentration. Comparison of the stability of LDHA-EVs, GNE140 treated LDHA-EVs and Ctrl-EVs under -80 °C for 2 weeks, suspended in pH 5.5 solution for 12 hours. LDHA concentration was detected at 3 time points (n = 3). (F) The illustration of GNE140 treated EVs and co-cluture with organoids. (G) The bio-distribution of PKH-67 labeled EVs co-cultured with Organoids. Representative images captured at 48h. Green indicates PKH-67 labeled EVs, Dapi indicates cell nucleus. (H) Statistics of percentage areas of PKH-67 labeled EVs/organoids. ns, non-significance. (I) 10µg Ctrl-EVs, LDHA enriched EVs, GNE140 were added to the media for 1 week and bright light, H&E. Blank, control group without adding EVs. Scale bars: 500µm. (J) multi-immunofluorescence staining for the Ki-67/SOX2/Nestin and HIF-1α/SOX2/Nestin in the organoids, blank, control group without adding EVs. scale bars: 200 µm (n = 8). (K-M) Statistics of diameter, Ki-67/SOX2/Nestin positive cells percentage, HIF-1α/SOX2/Nestin positive cells percentage, respectively. Blank, control group without adding EVs. Data are presented as means ± SD.; *, *p* < 0.05; **p < 0.01; ***p < 0.001. (N) HIF-1α/SOX2/Nestin were detected by western blotting, respectively. GAPDH were used as loading control. (O) Cell-counting kit-8 assay. Organoids were seeded in 96-well plates. 10µg Ctrl-EVs, LDHA enriched EVs, GNE140 were added to the media. Blank, control group without adding EVs. Proliferation activity was measured by CCK-8 assay once a day within 1 week. *, *p* < 0.05, **p < 0.01.

We investigated the role of exosomal LDHA by utilizing patient-derived GBOs, which were cu-cultured with engineered LDHA-enriched extracellular vesicles (LDHA-EVs). GBOs were generated using a non-disruptive method to preserve the original tumor architecture, as illustrated in the workflow (**Figure 5A**). LDHA-EVs were engineered by transducing U87-MG cells with LDHA-encoding lentivirus, followed by EV isolation (**Figure 5B**). Western blot analysis revealed significantly higher LDHA content in LDHA-EVs than in control EVs (**Figure 5C**). Characterization of these EVs confirmed their typical morphology, size distribution (**Figure 5D**). LDHA concentration within EVs were measured. Our data demonstrated no significant variation in LDHA levels after EVs were stored at -80 °C for 2 weeks while LDHA inhibitor GNE140 effectively suppress exosomal LDHA concentration (*p* < 0.01, **Figure 5E**). Subsequently, GNE140-treated EV particles were isolated and incubated with GBOs, as illustrated in the experimental design (**Figure 5F**).

Firstly, we have conducted GBO treatment experiments by using PKH67-labeled EVs for 48 h. The results indicated that approximately 60-70% of the EVs were distributed within the GBOs at 48 h. These distributions were observed to be across all the groups, including Ctrl-EVs, LDHA-EVs, and LDHA-EVs+GNE140 group, and there were no significant differences between the groups (**Figure 5G-H**). Furthermore, GBOs were co-cultured with control EVs, LDHA-EVs, or GNE140-treated LDHA-EVs for 7 days. Histological analysis confirmed that GBOs maintained the tumor characteristics (**Figure. 5I**). Immunofluorescence analysis revealed that LDHA-EVs significantly increased the proportion of cells expressing stem cell and proliferation markers (Ki67, SOX2, Nestin, and HIF-1α) in organoids compared to controls (**Figure 5J-M**). Besides, western blotting indicated the increased expression of SOX2, Nestin, and HIF-1α co-cultured with LDHA-EVs, and the effects reversed with GNE140 treatment (**Figure 5N**). Additionally, cell counting kit 8 assay further confirmed the higher proliferation ability of organiods treat with LDHA-EVs, compared to the other groups (**Figure 5O**). Conversely, GNE140-treated LDHA-EVs exhibited the opposite effect. These findings demonstrated that LDHA-EVs enhance the stemness phenotype within organoids, suggesting that LDHA-EVs potentially contribute to tumor stemness and progression.

### LDHA-enriched EVs promote metabolic reprogramming and lactate production

**Figure 6:**
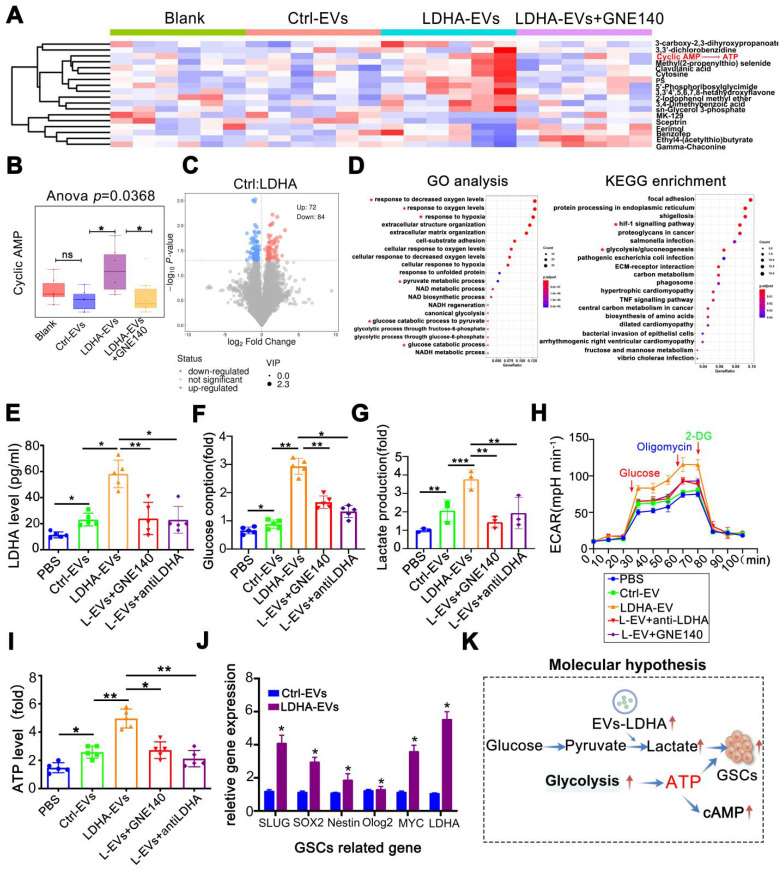
** LDHA-enriched EVs promote metabolic reprogramming and lactate production.** (A) Metabolomics of organoids and heatmap indicated markedly upregulated metabolite in groups of blank, Ctrl-EVs, LDHA-EVs and LDHA-EVs+GNE140. (B) cAMP (cyclic adenosine monophosphate) level analysis. (C) Differential metabolites in groups of Ctrl-EVs and LDHA-EVs. (D) Go analysis and KEGG analysis, the red asterisk represents the pathway of concern. Intracellular LDHA level (E) Glucose consumption (F) Relative Lactate level (G) ECAR rate (H) and ATP level (I) in the GBM organoids incubated with PBS, 10 µg Ctrl-EVs, LDHA-EVs, LDHA-EVs treatment with GNE140 and neutralizing antibody, respectively. (J) mRNA level of GSCs associated gene levels in the spheres incubated with LDHA-EVs and Ctrl-EVs (n = 3). (K) Schematic of molecular hypothesis. Data are presented as means ± SD. *, *p* < 0.05; **p < 0.01; ***p < 0.001.

Next, when metabolomic analysis was performed on GBOs, heatmaps revealed significant metabolic alterations with LDHA-EV treatment (**Figure 6A**). Notably, cAMP levels, related to ATP and the citric acid cycle (TCA) were significantly elevated in the LDHA-EV group. This effect was reversed by GNE140 (*p* = 0.0368) (**Figure 6B**). Differential metabolites are shown in **Figure 6C**. Pathway analyses highlighted the enrichment of glycolysis, pyruvate metabolism, and glucose consumption pathways (**Figure 6D**). To investigate whether LDHA-EVs mediated glycolysis, we detected glycolytic molecules in EV co-cultured organoids. Our data showed that LDHA-EVs significantly increased intracellular LDHA levels compared to the control-EV group. This effect was attenuated by LDHA inhibition by using GNE140 and a neutralizing LDHA antibody, indicating effective delivery of the LDHA enzyme by EVs (**Figure 6E**). Furthermore, LDHA-EVs caused higher glucose consumption and lactate levels in organoids (**Figure 6F-G**) attenuated by LDHA inhibitors. Seahorse assays showed elevated ECAR in LDHA-EV treated groups, which were reduced upon LDHA inhibition (**Figure 6H**). Consistent with enhanced glycolysis, LDHA-EVs increased basal ATP levels, an effect reversed with decreased LDHA activity (**Figure 6I**). qPCR analysis revealed up-regulation of stemness-associated genes after LDHA-EV uptake (**Figure 6J**), confirming that LDHA induced a stem cell-like phenotype. These results supported a potential molecular mechanism whereby EVs deliver LDHA enzyme, enhancing glycolysis and lactate production. This metabolic reprogramming potentially contributes to GBM stemness.

### Alleviating LDHA activities in circulating EVs potentially reduces GBM progression

**Figure 7:**
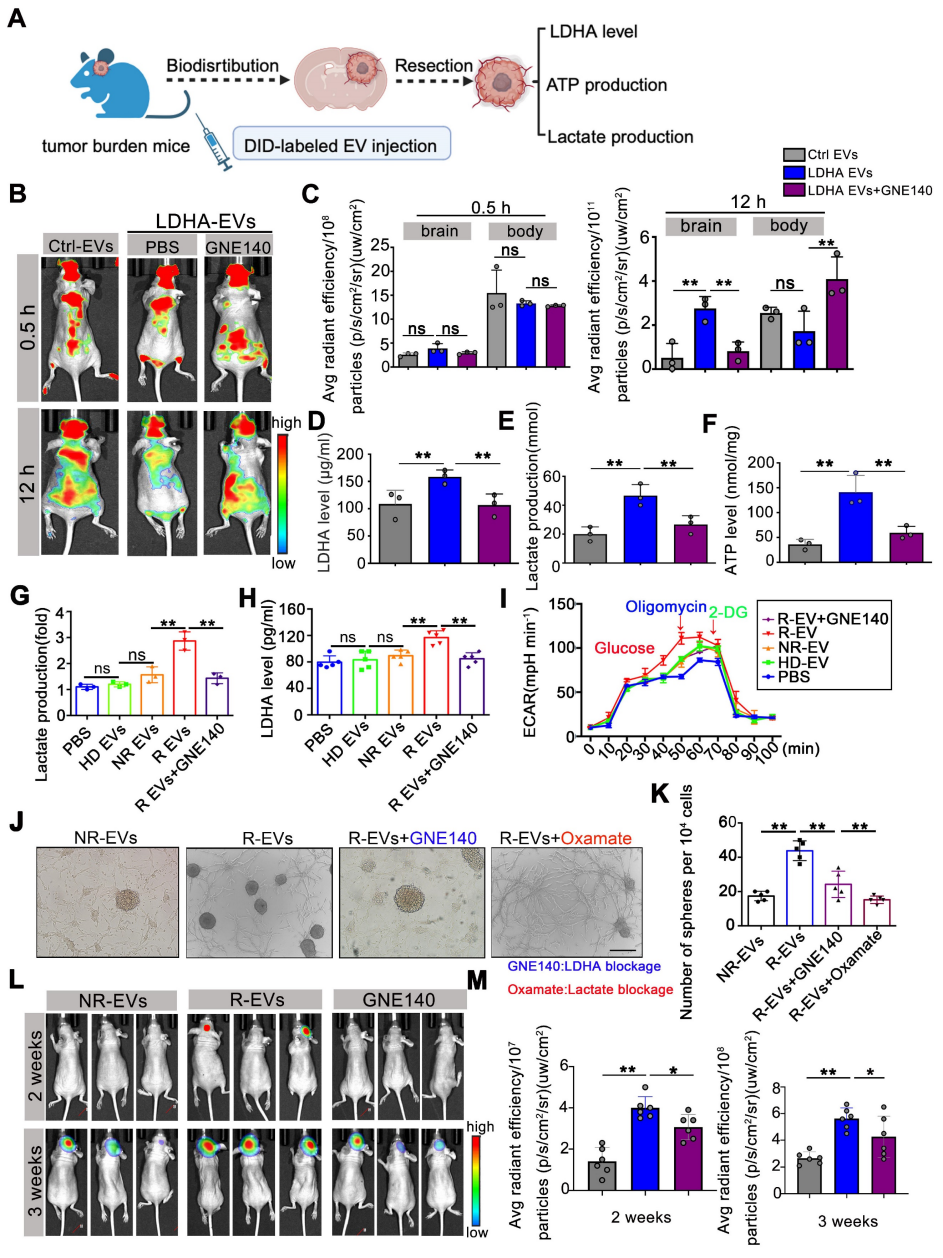
** Alleviating LDHA activities in circulating EVs potentially reduces GBM progression.** (A) Workflow of EVs injection and tissues detection. (B) DID labeled EV fluorescent in the mice *i.v.* injected Ctrl-EVs, LDHA-EVs, and GNE140 treated LDHA-EVs at 0.5 h and 12 h. (C) Statistics of fluorescence in the brain and body of mice at 0.5 h and 12 h, respectively. ATP level (D) lactate production (E) and LDHA level (F) of intracranial tumor tissues after EV administration. Intracellular Lactate level (E) LDHA level (F) and ECAR rate (G) in the spheres incubated with PBS, 10 µg plasma EVs (HD-EVs, NR-EVs, R-EVs and R-EVs pretreat with GNE140), respectively. (J) GSCs formation assay after uptake of plasma EV and blocking of LDHA with GNE140 or blocking lactate activities with oxamate, respectively. Scale bars: 200µm. (K) Statistics of GSC-associated spheres numbers. (L) Representative images of tumor growth with plasma EV administration, at 2 weeks and 3 weeks. (M) Statistics of tumor burden *, *p* < 0.05; **p < 0.01; ***p < 0.001.

To investigate the effect of exosomal LDHA activity on tumor progression, we intravenously administered Ctrl-EVs, LDHA-EVs, and GNE140-treated EVs to U87-MG tumor-bearing mice and assessed EV distribution, tumor progression, and intracranial ATP, lactate, and LDHA levels (**Figure 7A**). At 12 h post-injection, mice administered with LDHA-EVs exhibited markedly enhanced intracranial fluorescence signals, which were attenuated by GNE140, suggesting that inhibition of LDHA activity in EVs reduced their homing capacity to the brain (**Figure 7B-C**). Furthermore, LDHA-EV administration markedly increased LDHA, lactate, and ATP levels in the local tumor region, while GNE140 reversed these effects (**Figure 7D-F**).

To investigate the clinical potential, EVs derived from recurrent GBM patient plasma (R-EVs) were treated with GNE140 and added to organoids. Our data showed that R-EVs significantly increased intracellular LDHA levels (*p* < 0.01) and lactate production in spheres (*p* < 0.01), while the elevated ECAR rate was attenuated by GNE140 (*p* < 0.01) (**Figure 7G-I**). The R-EV-induced increase in sphere formation was suppressed *in vitro* by GNE140 (an LDHA inhibitor) (*p* < 0.01) and oxamate (a lactate inhibitor) (*p* < 0.01), indicating that blockade of LDHA and lactate production contributes to alleviating the GSC phenotype (**Figure 7J-K**). R-EVs promoted tumor growth at 2-3 weeks compared to the non-recurrent group after EV administration (2 weeks, *p* < 0.01, 3 weeks, *p* < 0.01). This effect was reversed when LDHA activity in R-EVs was inhibited by GNE140 pretreatment (*p* < 0.05) (**Figure 7L-M**). These data suggested that reducing LDHA activity in circulating EVs potentially inhibited tumor growth. Our findings indicated that targeting LDHA-carrying EV subpopulations prevent GBM progression, offering a novel therapeutic strategy for this aggressive malignancy.

### Circulating LDHA-EVs correlate with a poor outcome of recurrent GBM and serve as a candidate non-invasive biomarker

**Figure 8:**
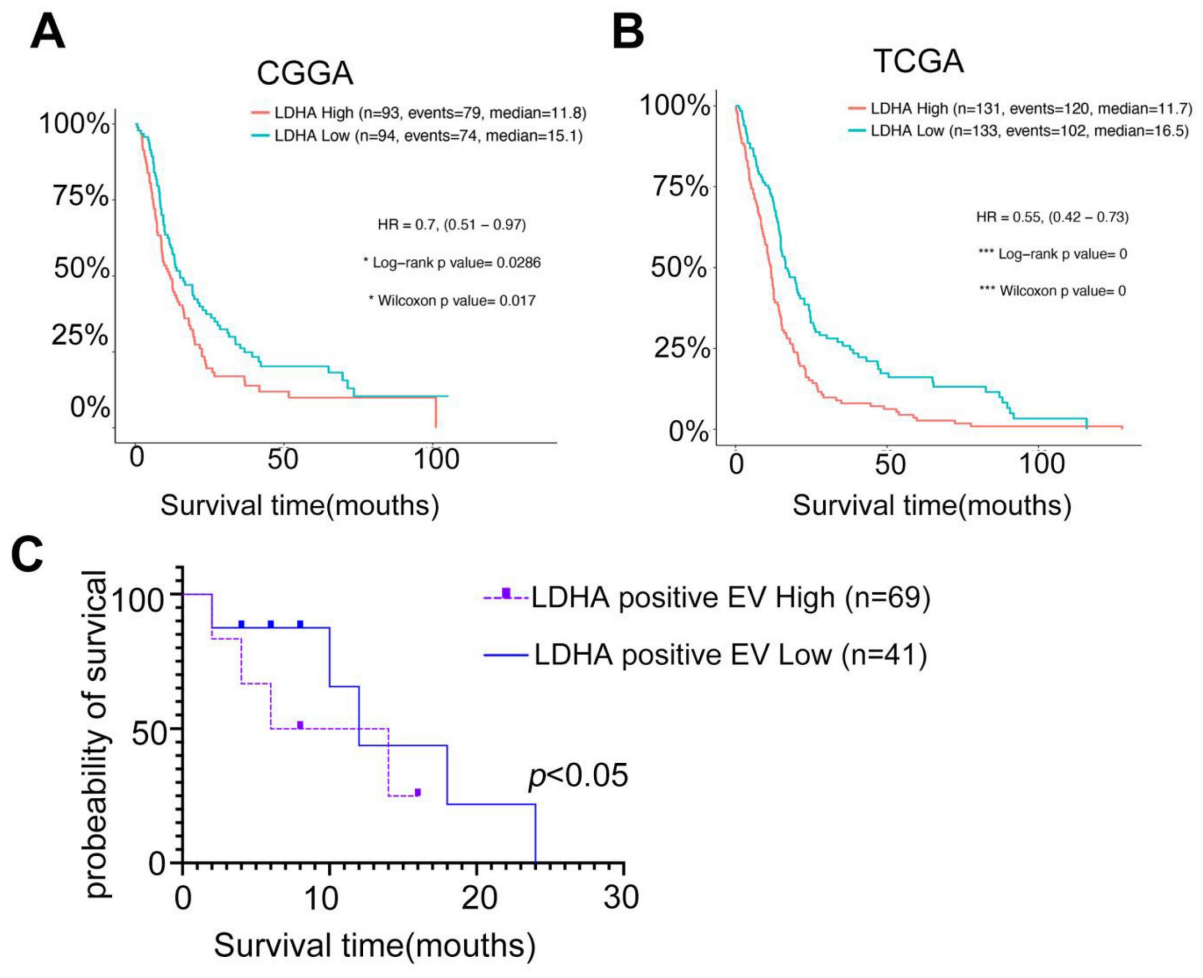
** Circulating LDHA-EVs correlate with a poor outcome of recurrent GBM and serve as a candidate non-invasive biomarker.** (A) Kaplan-Meier survival analysis of the expression levels of LDHA in GBM patients based on the CGGA database. (B) Kaplan-Meier survival analysis of the expression levels of LDHA in GBM patients based on the TGGA database. (C) Survival analysis of the levels of LDHA positive EVs in recurrent GBM patients based on the cohorts.

Studies have demonstrated LDHA's potential as a promising biomarker for GBM prognosis in clinical settings. Analyses of The Cancer Genome Atlas (TCGA) and Chinese Glioma Genome Atlas (CGGA) databases revealed that GBM patient prognosis was negatively correlated with LDHA expression (**Figure 8A-B**). Furthermore, cohort studies indicated that the levels of LDHA-positive EVs in recurrent GBM patients were inversely associated with patient survival (**Figure 8C**). Our study demonstrated that LDHA enzymes encapsulated in plasma extracellular vesicles activate glioblastoma stemness by enhancing glycolysis. We highlighted the potential of LDHA as a signal transducer transmitted via EVs to promote cancer aerobic glycolysis and the GSC phenotype. This finding provides novel insights for monitoring recurrent GBM and predicting GBM sensitivity to radio-chemotherapy using liquid biopsy techniques.

## Discussion

GBM's aggressiveness, treatment resistance, and recurrence appear to originate from a low abundance subpopulation of GSCs within tumor cells, which show functional properties such as low proliferative activity, self-renewal, and multipotency 30-33. Metabolic reprogramming is the hallmark of GBM progression relying on glycolysis and accumulating lactate significantly, resulting in an unfavorable prognosis 34-35. In the tumor center, hypoxia renders tumor cells to undergo glycolysis, while aerobic glycolysis may also be induced at tumor margins away from the hypoxic central areas 36. Histopathology confirmed the presence of many infiltrating tumor cells in the brain tissue surrounding the tumor with marked hypoxia and stemness markers, implying that there may be strong metabolic remodeling.

Previous studies have focused on excess lactate production, enhanced hypoxia, and stemness of GBM tissues, however, the involvement of peripheral EV cargo in GBM recurrence was not reported. Our data demonstrated that the EV-facilitated lactate-ATP-cAMP cycle contributes to metabolic reprogramming and GSC form/action. We provide novel insights into the potential function of circulating EV cargo to metabolize excess glucose. One recent study pointed out that LDHA upregulated C-C motif chemokine ligand 2 (CCL2) and CCL7 through the ERK-YAP-STAT3 signaling axis to recruit macrophages into the tumor microenvironment. The infiltrating macrophages produced LDHA-containing EVs to promote GBM cell metabolic remodeling, proliferation, and survival 37. In our study, we mentioned that EVs from infiltrating tumor cells in the brain tissue surrounding exhibited strong stemness may also release LDHA enriched EVs and regulated by TMZ/radiotherapy post-operation (**Figure 4**).

Furthermore, two new studies have indicated that lactate-induced post-translational modifications regulate homologous recombination and promote chemoresistance in cancers 38-39. In addition to metabolic regulation, non-metabolic functions and the relationship between plasma EVs and lactate accumulation or lactation modification in the relapsed GBMs are also noteworthy. Other investigators have demonstrated elevated plasma EV levels with a high protein load in primary and recurrent GBM patients 16. EVs may provide an effective cargo delivery system to target GBMs due to their ability to cross the BBB and enter the glioblastoma micro-environment 39. The current understanding of the origin and functions of cancer-derived EVs might enable their exploitation for anticancer therapy 41.

It has been reported that EVs from mouse and human lung-, liver-, and brain-tropic tumor cells fuse preferentially with resident cells in different organs. EV integrins could predict organ-specific metastasis, demonstrating their capacity for long-distance communication 42. However, identifying tumour-intrinsic properties and/or drivers of the crosstalk between tumour cells and the brain micro-environment that can be targeted is critical. Previous study indicated that, small EVs (sEVs) derived from metastatic melanoma cell lines were enriched in nerve growth factor receptor (NGFR, p75NTR), could spread through the lymphatic system, and were taken up by lymphatic endothelial cells, supporting lymph node metastasis 43. David lyden *et al.* demonstrated that exosomal CEMIP induces a pro-inflammatory state in the brain vascular niche that supports brain metastatic colonization 44. These data support the notion that the combination of biophysical properties and surface proteins influences sEV dissemination. Whether circulating and EV particles reach the target organ by passive or active mechanisms is debatable. In our study, we found that EVs from glioma cells and recurrent GBM patients' plasma displayed elevated ATP and lactate levels after uptaking by recipient cells and accelerated homing effects when they spread through the blood. Based on our studies, along with findings from the David and prior research on the LDHA gene, we propose that as brain is an organ with heightened lactate consumption, and GBM typically exhibit elevated levels of hypoxia and lactation consumption. Extracellular vesicles carrying LDHA enzyme preferentially accumulate in tumor regions and providing ATP and enhanced lactate level may an important reason for the EV brain-targeting and contribute to the tumor progression. Additionally, the underlying genetic regulatory mechanisms require further elucidation. However, the LDHA-EV injection rescued LDHA inhibitor treatment. The observation that inhibitors block EV homing provides novel insights into many therapeutic opportunities to target LDHA/LDHB in glioma treatment.

The standard method for monitoring the treatment response of radio-chemotherapy is clinical evaluation and magnetic resonance imaging (MRI) from two to six months. However, 20% of patients treated with TMZ chemoradiotherapy show pseudo-progression 42-43 which is difficult to distinguish from actual progression. Only surgery followed by pathological investigation can verify the progressive state, which is unnecessary if the lesions are not progressive. Therefore, a less time-consuming and non-invasive method for treatment monitoring is needed. In this context, blood-based biopsy seems promising. Several methods have been proposed to monitor liquid-based alterations, including circulating tumor cells (CTCs) or alterations detected in cerebrospinal fluid (CSF) 45-46. Due to their ability to pass through the BBB, EVs can be potential markers for GBM Our study found elevated levels of LDHA-enriched EVs in the plasma with GBM relapse, associated with glycolysis, poor chemotherapeutic response, and shorter survival of patients. These observations implied that LDHA-enriched EVs in plasma might be a valuable blood-based biopsy for verifying the pseudo-progression and monitoring the treatment response and progression of GBM. It will also be essential. to determine whether progression could be detected by LDHA-enriched EVs using blood-based biopsy before the clinical and/or radiological evidence.

Our study highlights the potentially crucial role of circulating EVs after GBM resection. These data provide evidence for monitoring recurrent GBMs. A better understanding of LDHA-enriched EV subpopulation as a potential 'metabolic switch' is needed to function as a non-invasive biomarker and therapeutic target, providing novel insights for GBM diagnosis and preventing recurrence in future clinical translation**.**

## Supplementary Material

Supplementary materials and methods, figures and table.
