# Supplementary material for: Plasma extracellular vesicles from recurrent GBMs carrying LDHA to activate glioblastoma stemness by enhancing glycolysis
Source: Theranostics. 2025 Feb 26;15(8):3655–72. doi: 10.7150/thno.102014 (PMC11905145; doi:10.7150/thno.102014)
Supplement: Supplementary file 1 — Supplementary materials and methods, figures and table. [file thnov15p3655s1.pdf]

Table 1 Clinical information of GBM patients in this study.

**Number of cases**	265
Non-recurrence	84
Recurrence	181
**Median age**	55.5 (19-75)
**Age(years)**	
0-50	148
>50	117
**Gender**	
Male	137
Female	128
**IDH mutation**	
IDH Wt	252
IDH mutant	13

Supplementary Figure 1 Transmission electron microscopy.

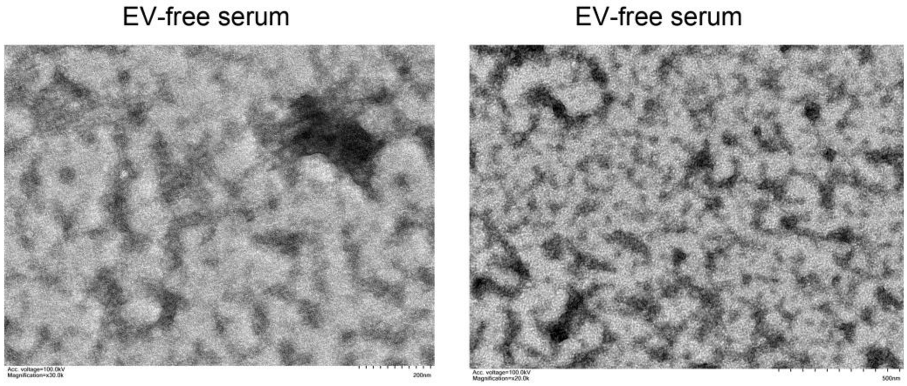

Transmission electron microscopy detection of EVs from EVs depleted serum. Bars represents 200 nm (left) and 500 nm (right).

Supplementary Figure 2 Immunostaining.

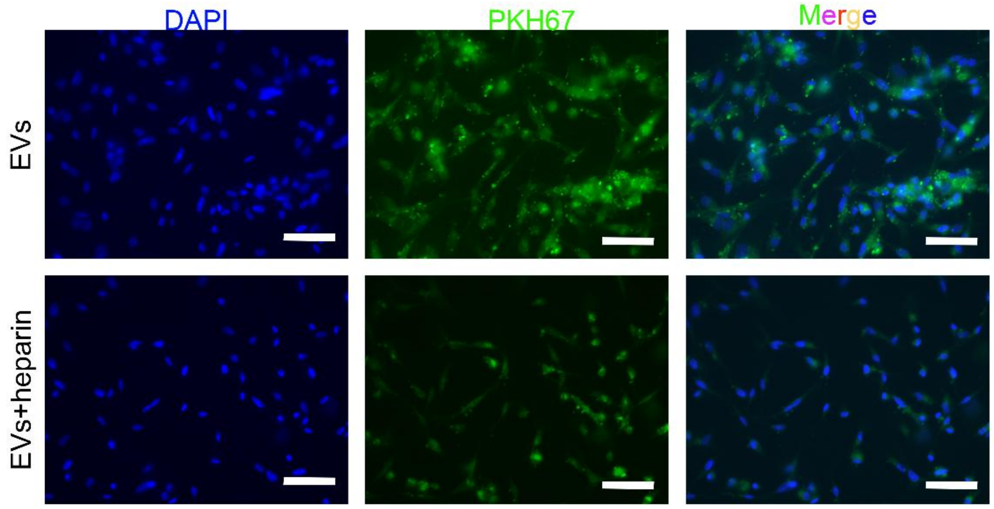

Immunostaining of cells co-cultured with EVs treated with 2μg/μl heparin for 48 h. Bars represents 100 μm.

Supplementary Figure 3 Representative images of tumor area and adjacent brain tissues.

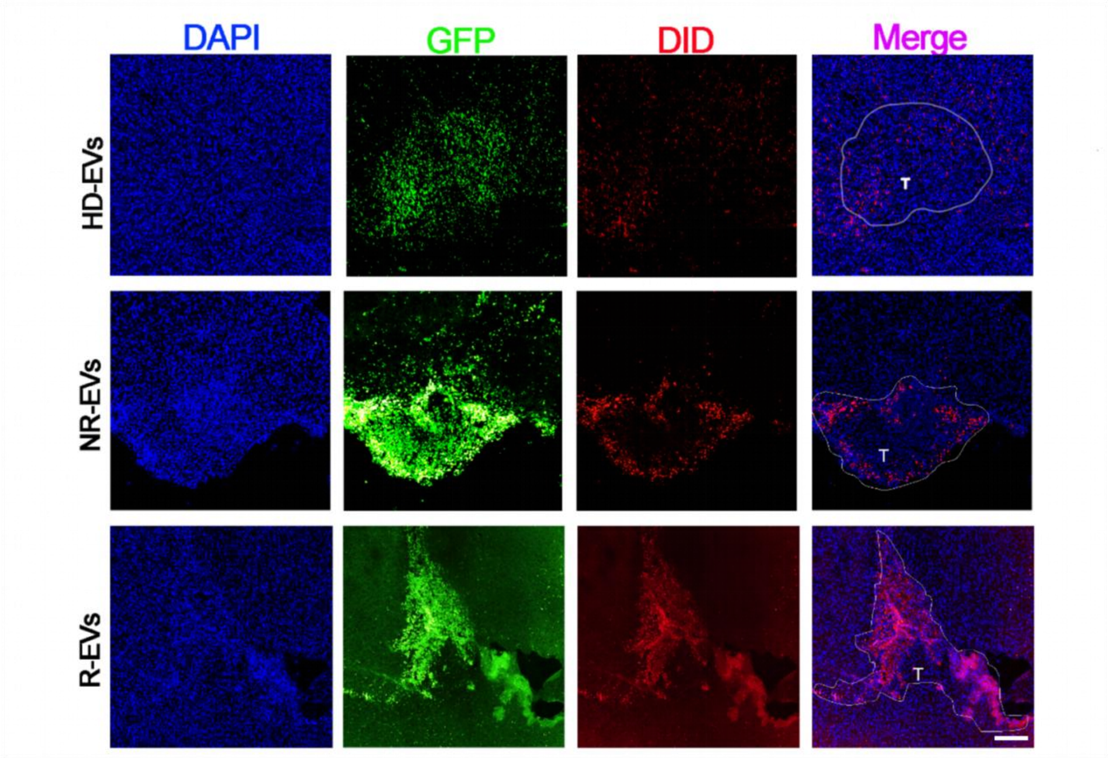

Representative images of tumor area and adjacent brain tissues. Brain sections were obtained from intracranial U87-Lentivirus-GFP tumor-bearing mice treated with 30 μg of EVs (HD-EVs, NR-EVs, or R-EVs). The brain sections were collected 12 h after EV injection. GFP (green)

indicates the tumor area, DiD (red) shows the distribution of EVs, and DAPI (blue) labels the nuclei. T, represents the tumor area. Bar represents 20 μm.

Supplementary Figure 4 Histological analysis.

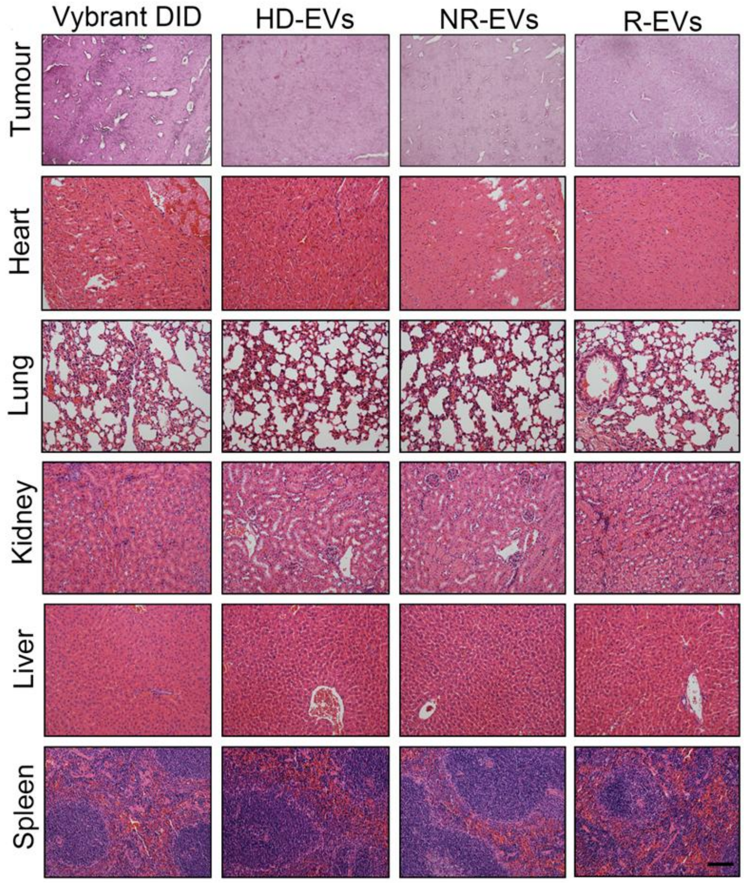

Histological analysis showed that pl-EVs injection does not lead to any histological changes in typical organs. Bar represents 20 μm.

Supplementary Figure 5 Statistics of relative (Rel.)

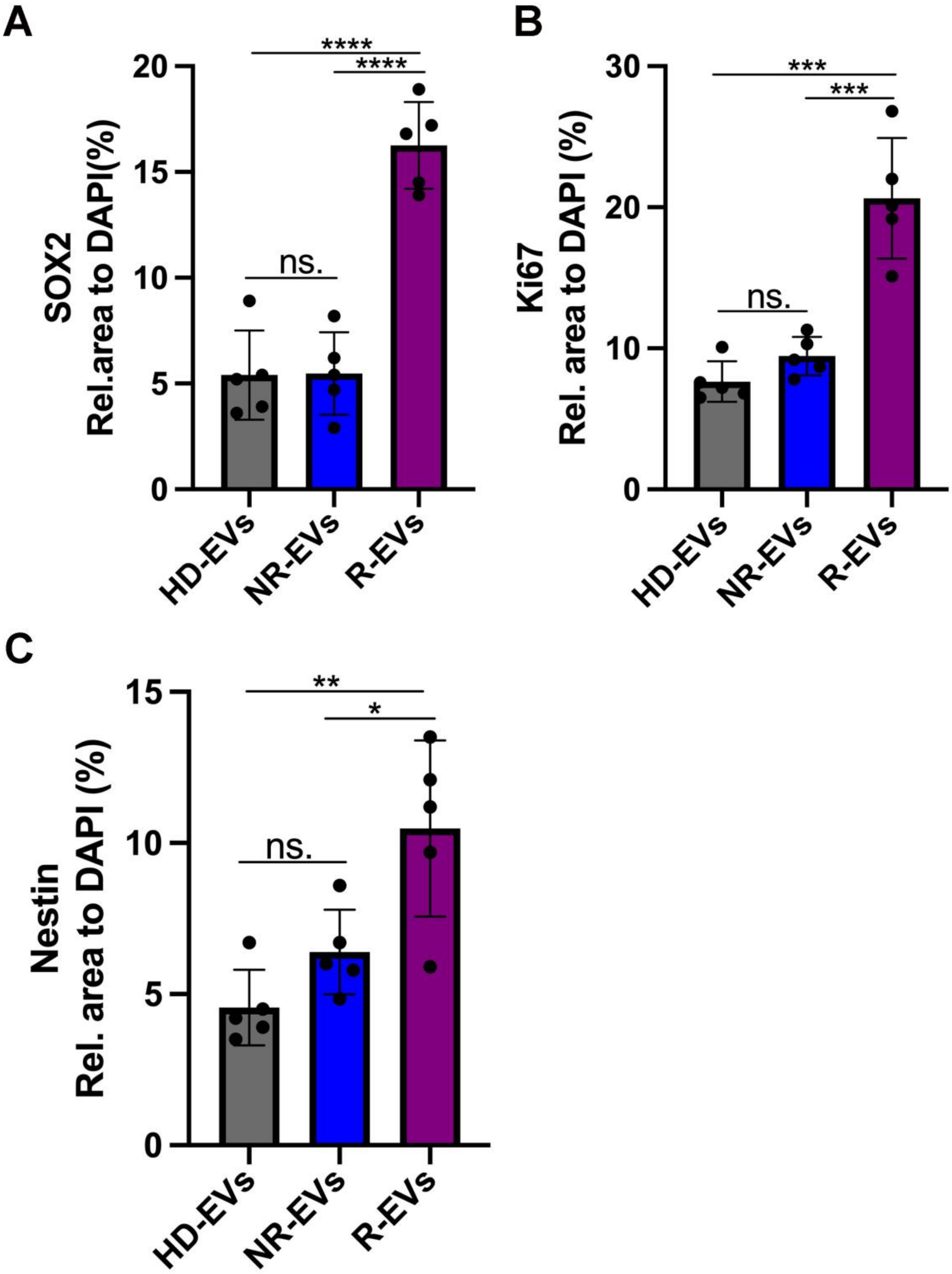

Statistics of relative (Rel.) SOX2 to DAPI(%) (A), relative (Rel.) Ki67 to DAPI(%) (B), relative (Rel.) Nestin to DAPI(%) (C).

(*, $p < 0.05$,**,$p < 0.01$,***, $p < 0.001$,****, $p < 0.0001$, ns, non-significance).

Table 2 Ingenuity canonical pathways.

Ingenuity Canonical Pathways	Acc.no.	Entry name	Protein description	Expr Fold Change	-log (P-value)
**1.Pyruvate Fermentation to Lactate**					**3.76**
	P00338	LDHA	lactate dehydrogenase A	2.381	
	P07195	LDHB	lactate dehydrogenase B	1.826	
**2.Actin Cytoskeleton Signaling**					**4.47**
	P23528	CFL1	cofilin 1	-1.284	
	P02751	FN1	fibronectin 1	-1.493	
	P06396	GSN	gelsolin	-1.33	
	P18428	LBP	lipopolysaccharide binding protein	1.31	
	P26038	MSN	moesin	-2.008	
	Q9Y490	TLN1	talin 1	1.413	
	P18206	VCL	vinculin	1.542	
**3.LXR/RXR Activation**					**4.91**
	P04114	APOB	apolipoprotein B	-1.458	
	P0C0L4	C4A/C4B	complement C4B	-1.389	
	P18428	LBP	lipopolysaccharide binding protein	1.31	
	Q9UHG3	PCYOX1	prenylcysteine oxidase 1	-1.37	
	P05109	S100A8	S100 calcium binding protein A8	1.214	
	P01009	SERPINA1	serpin family A member 1	1.456	
**4.Gluconeogenesis Ⅰ**					**5.46**
	P04075	ALDOA	aldolase, fructose-bisphosphate A	-1.21	
	P06733	ENO1	enolase 1	-1.253	
	P00558	PGK1	phosphoglycerate kinase 1	1.239	
**5.Glycolysis Ⅰ**	P04075	ALDOD	aldolase,fructose-bisphosphate A	-1.214	**5.53**
	P06733	ENO1	enolase I	-1.253	
	P00558	PGK1	phosphoglycerate kinase 1	1.239	
**6.Intrinsic Prothrombin Activation Pathway**	P03951	F11	coagulation factor XI	-1.441	**6.1**
	P00748	F12	coagulation factor XII	-1.488	
	P02675	FGB	fibrinogen beta chain	1.268	
	P02679	FGG	fibrinogen gamma chain	1.28	
	P07225	PROS1	protein S	-1.37	
**7.Extrinsic Prothrombin Activation Pathway**	P00748	F12	coagulation factor XII	-1.488	**6.29**
	P02675	FGB	fibrinogen beta chain	1.268	
	P02679	FGG	fibrinogen gamma chain	1.28	
	P07225	PROS1	protein S	-1.37	
**8.Complement System**	P02745	C1QA	complement C1qA chain	1.357	**6.4**
	P02746	C1QB	complement C1qB chain	1.394	
	P0C0L4	C4A/C4B	complement C4B	-1.389	
	P04003	C4BP4	complement 4 binding protein alpha	-1.366	
	P20851	C4BPB	complement 4 binding protein beta	1.598	
**9.Acute Phase Response Signaling**	P0C0L4	C4A/CAB	complement C4B	-1.389	**7.21**
	P04003	C4BPA	complement 4 binding protein alpha	-1.366	
	P20851	C4BPA	complement 4 binding protein beta	1.598	

	P02675	FGB	fibrinogen beta chain	1.268
	P02679	FGG	fibrinogen gamma chain	1.28
	P02751	FN1	fibrinogen 1	-1.493
	P02792	FTL	ferritin light chain	2.242
	P18428	LBP	lipopolysaccharide binding protein	1.31
	P05109	S100A8	S100 calcium binding protein A8	1.214
	P01009	SERPINA1	serpin family A member 1	1.456

Supplementary Figure 6. H&E staining and 1p19q confirmed by FISH of recurrence GBM patients.

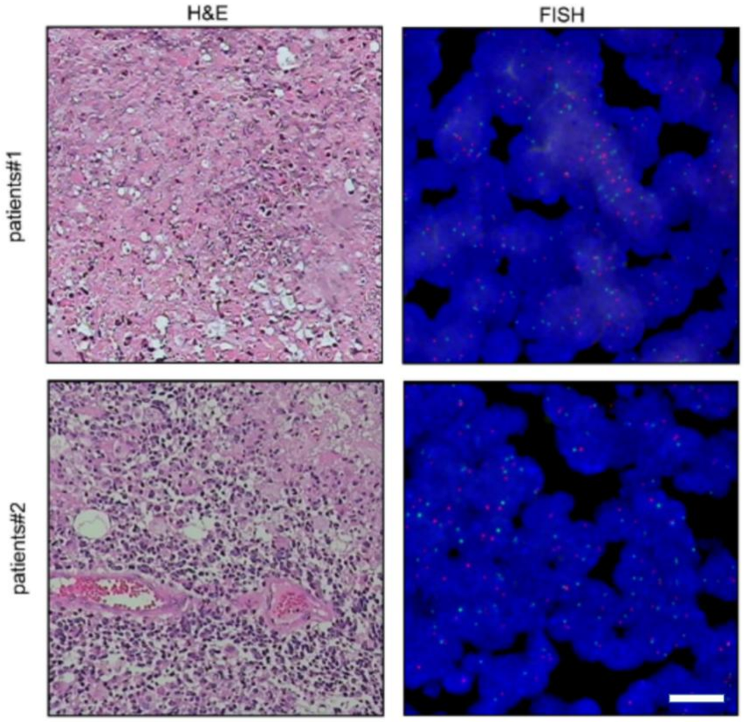

H&E staining. 1p19q confirmed by FISH of recurrence GBM patient#1 and patient#2. Colourful points represents the expression of 1p19q. Bar represents 50 μm.

Supplementary Figure 7. GSEA and KEGG of LDHA associated pathways.

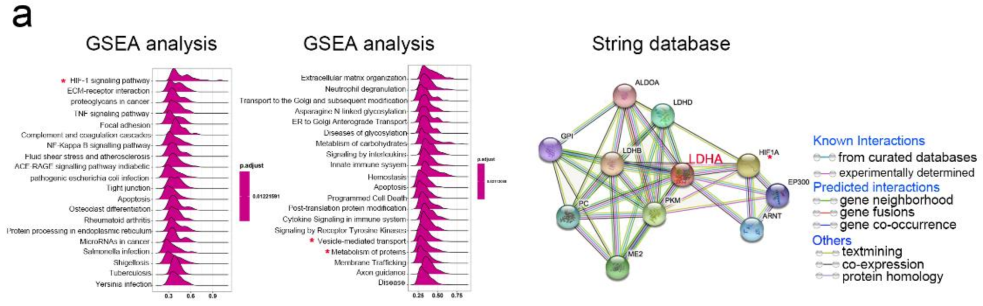

Supplementary Figure 8. Western blotting

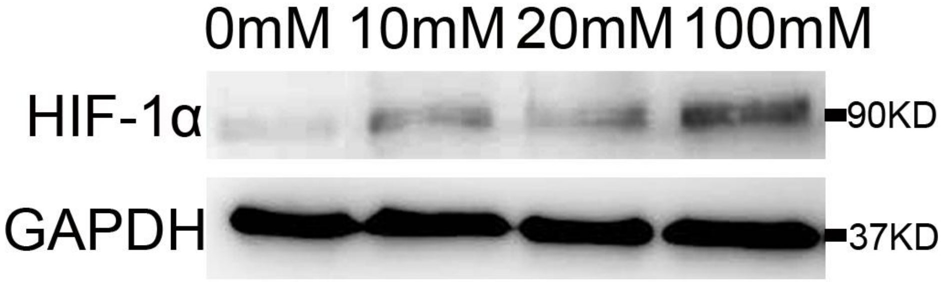

Western blotting detects of HIF-1α and GAPDH in U87-MG treated with conditioned $CoCl_2$ (0, 10, 20, 100 mM) for 48h.

Supplemental materials and methods

1.Antibodies.

Antibodies were as follows: CD63 (Abcam, ab217345), CD9 (Abcam, ab92726), TSG101 (Abcam, ab125011), Albumin (Abcam, ab207327), Calnexin (Abcam, ab152012), CD44 (biolegend, #397502), LDHA (For western blotting, Abcam, ab52488, For exo-counter, specific EV capture, Santa cruze, sc-137243, For LDHA partially blocking, Abcam, ab300637), HIF-1α (Abcam, ab179483), PHD2 (Cell Signaling Technology, #4835), GAPDH (sc365062, Santa Cruz Biotechnology), and β-Actin (sc-47778, Santa Cruz Biotechnology).

2.Animal study.

Orthotopic models were constructed by using U87-ctrl and U87-Luc with six-week-old female nude mice, as our previous reported [28]. All the animal experiments were approved by the responsible ethics committee of Nanfang hospital (#NFEC-2022-056).

3.IVIS Spectrum *In Vivo* Imaging.

Luciferin substrate (Abcam, ab145164) was diluted in DPBS (Sigma Aldrich, D8537)

at 15 μg/μl concentration and filtered with a 0.2 μm filter immediately. Luciferin substate were intra-peritoneally (*i.p.*) injected 30 mins before imaging with a 100μl dose per mouse. Mice were anesthetized 10 mins before detected on IVIS Spectrum I*n Vivo* Imaging System (AMI HTX, Spectral Instruments Imaging). DID dye were used (VybrantTM cell-labeling solutions, MP22885) to label and track EVs distribution, mice were examined in 0.5 h and 12 h and the organs were harvested at 24 h, detected with 665 nm emission wavelength.

4.Protein measurement of EV preparations.

The protein concentrations in the SEC fractions were measured with a BCA assay Kit (Beyotime BCA Protein Assay Kit ™) according to the manufacturer's instruction.

5.PKH67 labelled EVs uptake *in vitro*.

~10 μg EV samples labeled with 0.1 μM PKH67 (Sigma-Aldrich), incubated at 37 ℃ for 15 mins, and then centrifuged at 120,000 × g for 90 mins. The recipient cells were incubated with the PKH67 labeled EVs in the dark for 6 h, removed the supernatant and fixed with 4% paraformaldehyde (PFA) for 15 mins, as previous described [26], and then stained with DAPI and fluorophore-conjugated antibody to visualize the nucleus, Nestin and SOX2, respectively. Immunofluorescence images were taken with inverted confocal microscope (ZEISS LSM980, Germany).

6.Western blot.

~15 μg EVs samples were uploaded in 20-mM Tris-HCl 1% SDS and separated on 10% polyacrylamide gels. Molecular-weight-dependent protein separation by sodium dodecyl sulfate-polyacrylamide gel electrophoresis (SDS-PAGE), and transferred

onto PVDF membranes (Roche, Basel, Switzerland). Membranes were blocked for 1 h and incubated with primary antibodies and incubated with the corresponding secondary antibodies for 1 h at room temperature. EVs markers including TSG101, CD9, CD63, endoplasmic reticulum chaperone protein: Calnexin, HIF-1α, LDHA and PHD2 in cell lysates etc., as previous reported [28].

7.Spheroid identification.

~5μg EV samples were added to the medium with ~20,000 cells, and the observation was continued for ~1-2 weeks. The GSCs-like spheroids were visible at 3-5 days, and the number of spheroid were counted under the microscope (Leica DM IL LED, Leica), spheroid size was estimated using Spheroid Sizer. Immunofluorescence staining of Nestin and SOX2 was performed to identify GSCs phenotype.

8.TMZ-resistant cells model

We treated U87MG cells with a mild concentration of temozolomide (TMZ, Sigma-Aldrich, #85622-93-1) for 3-4 weeks until the cells proliferate normally, then increase the concentration of TMZ gradually and repeat the above culture process, and the same concentration of Dimethyl sulfoxide (DMSO, Sigma-Aldrich, #276855) was used as placebo control. From this panel until the cells tolerate the concentration of TMZ reached 1000 M/L, the temozolomide-sensitive placebo control cells (U87MG-P) and the TMZ-resistant cells (U87MG-R) were generated. U87MG cells were seeded into 60 mm culture dishes (3×10^5 cells/dish) in 5 ml of medium and incubated for 48 h. After incubation, the cells were exposed to X-radiation at doses of 4Gy.

9.Isolation of cell culture derived EVs.

Cell-conditioned media (600-1200 ml) 80% confluent U87-Ctrl and U87-LDHA cells were cultured at (~2 × 10^5/ml) concentration DMEM (Gibco) containing 10% EV-depleted FBS (Gibco). To isolate EVs from U87-Ctrl and U87-LDHA cells, the conditioned medium centrifuged at 300 × g for 10 mins and at 2000 × g for 20 min to eliminate cells and cell debris and the larger EVs. Supernatants were then centrifuged at 16,500 × g (Type 45 Ti rotor, Beckman Coulter) for 20 mins at 4 ℃ and then at 118,500 × g (Type 45 Ti rotor, Beckman Coulter) for 2.5 h at 4 ℃. The pellets were mixed and dissolved in PBS and then stored at −80 ℃.

10.Metabolomics of the organoids.

Vanquish (Thermo Fisher Scientific) ultra-high performance liquid chromatograph were used, via Waters Acquity Uplchss T3 (2.1 mm × 100 mm, 1.8 μm) liquid chromatographic separation of target compounds. Liquid chromatography phase A is an aqueous phase containing 5 mmol/L ammonium acetate and 5 mmol/L acetic acid, phase B is acetonitrile, 2 μL sample were uploaded at 4 °C. Xcalibur (version 4.4, Thermo) were used to analysis raw data.
